# Sand fly synthetic sex-aggregation pheromone co-located with insecticide reduces the incidence of infection in the canine reservoir of visceral leishmaniasis: A stratified cluster randomised trial

**DOI:** 10.1371/journal.pntd.0007767

**Published:** 2019-10-25

**Authors:** Orin Courtenay, Erin Dilger, Leo A. Calvo-Bado, Lidija Kravar-Garde, Vicky Carter, Melissa J. Bell, Graziella B. Alves, Raquel Goncalves, Muhammad M. Makhdoomi, Mikel A. González, Caris M. Nunes, Daniel P. Bray, Reginaldo P. Brazil, James G. C. Hamilton

**Affiliations:** 1 Zeeman Institute and School of Life Sciences, University of Warwick, Coventry, United Kingdom; 2 School of Life Sciences, Institute of Science & Technology in Medicine, Keele University, Keele, Staffordshire, United Kingdom; 3 Division of Biomedical and Life Sciences, Faculty of Health and Medicine, Lancaster University, Bailrigg, Lancaster, Lancashire, United Kingdom; 4 Faculdade de Medicina Veterinária, Universidade Estadual Paulista (UNESP), Araçatuba, São Paulo, Brazil; 5 Laboratório de Doenças Parasitárias, Instituto Oswaldo Cruz, Rio de Janeiro, Brazil; Universidade Federal de Minas Gerais, BRAZIL

## Abstract

**Objective:**

To evaluate the efficacy of a synthetic sex-aggregation pheromone of the sand fly vector *Lu*. *longipalpis*, co-located with residual insecticide, to reduce the infection incidence of *Leishmania infantum* in the canine reservoir.

**Methods:**

A stratified cluster randomised trial was designed to detect a 50% reduction in canine incident infection after 24 months in 42 recruited clusters, randomly assigned to one of three intervention arms (14 cluster each): synthetic pheromone + insecticide, insecticide-impregnated dog collars, or placebo control. Infection incidence was measured by seroconversion to anti-*Leishmania* serum antibody, *Leishmania* parasite detection and canine tissue parasite loads. Changes in relative *Lu*. *longipalpis* abundance within households were measured by setting three CDC light traps per household.

**Results:**

A total 1,454 seronegative dogs were followed-up for a median 15.2 (95% C.I.s: 14.6, 16.2) months per cluster. The pheromone + insecticide intervention provided 13% (95% C.I. 0%, 44.0%) protection against anti-*Leishmania* antibody seroconversion, 52% (95% C.I. 6.2%, 74·9%) against parasite infection, reduced tissue parasite loads by 53% (95% C.I. 5.4%, 76.7%), and reduced household female sand fly abundance by 49% (95% C.I. 8.2%, 71.3%). Variation in the efficacy against seroconversion varied between trial strata. Equivalent protection attributed to the impregnated-collars were 36% (95% C.I. 14.4%, 51.8%), 23% (95% C.I. 0%, 57·5%), 48% (95% C.I. 0%, 73.4%) and 43% (95% C.I. 0%, 67.9%), respectively. Comparison of the two interventions showed no statistically consistent differences in their efficacies; however, the errors were broad for all outcomes. Reductions in sand fly numbers were predominant where insecticide was located (chicken and dog sleeping sites), with no evidence of insecticide-induced repellence onto humans or dogs.

**Conclusion:**

The synthetic pheromone co-located with insecticide provides protection particularly against canine *L*. *infantum* parasite transmission and sand fly vector abundance. The effect estimates are not dissimilar to those of the insecticide-impregnated collars, which are documented to reduce canine infection incidence, human infection and clinical VL disease incidence, in different global regions. The trialled novel lure-and-kill approach is a low-cost potential vector control tool against ZVL in the Americas.

## Author summary

The predominant sand fly vector of the intracellular parasite *Leishmania infantum*, that causes human and canine visceral leishmaniasis in the Americas, is *Lutzomyia longipalpis*. Dogs are the proven reservoir. Vector control tools to reduce transmission suited to this predominantly exophilic vector are lacking. Insecticide-impregnated dog collars protect dogs against infectious bites from sand fly vectors, and result in reductions of new infections in both dogs and humans. However, collars are costly for endemic communities, and alternative approaches are needed. Recently the bulk synthesised sex-aggregation pheromone of male *Lu*. *longipalpis* was shown to attract large numbers of conspecific females to lethal pyrethroid insecticides, indicating the potential for use in a vector control application. This study, conducted in Brazil, evaluated the efficacy of this novel lure-and-kill approach to reduce seroconversion and infection incidence with *L*. *infantum* in the canine reservoir, in addition to measuring its impact on household abundance of *Lu*. *longipalpis*. Deployed in 14 stratified clusters, the outcomes were compared to those attributed to insecticide impregnated collars fitted to dogs in another 14 clusters; each intervention was compared to 14 clusters that received placebo treatments. The beneficial effects of the lure-and-kill method were most noticeable on confirmed infection incidence and clinical parasite loads, and in reducing sand fly abundance. The overall effect of the two interventions were not statistically dissimilar, though the confidence intervals were broad. We conclude that the novel low-cost lure-and-kill approach should be added to the vector control toolbox against visceral leishmaniasis in the Americas.

## Introduction

Sustainable control of arthropod vectors to reduce infectious disease transmission represents a major challenge confronting public health programmes[1]. Standard approaches such as indoor residual spraying of insecticides (IRS) or insecticide treated nets (ITNs), are most effective against insecticide-susceptible vector populations that are endophilic and/or bite when hosts at risk are under ITNs e.g. as against *Anopheles gambiae*[2]. Suboptimal insecticide-based vector control occurs when contact rates with insecticide treated surfaces by susceptible vectors is less frequent[3], as expected following IRS/ITN campaigns against exophilic vectors. One potential solution is to lure biting vectors to strategically placed insecticide using attractant semiochemicals (kairomones and pheromones). Specific insect pheromones mediate conspecific mating (sex), aggregation, oviposition or invitation behaviour[4]. In the agricultural sector, integrated pest management programs deploy pest pheromones to monitor and reduce pest populations and disrupt pest mating aggregations, with the aim to limit crop yield loss, environmental damage, and insecticide use[4–6]. In contrast, whilst some pheromones produced by vectors of public or veterinary health importance have been identified e.g.[7], they appear to be absent or not characterised in many of the most important human and animal disease vectors. Indeed, to our knowledge, there are no published studies that have tested the efficacy of a vector pheromone to reduce infection or disease incidence.

One important vector species that produces a large amount of sex-aggregation pheromone is *Lutzomyia longipalpis* (Diptera: Psychodidae). This is the principal vector of *Leishmania infantum* (Kinetoplastida: Trypanosomatidae) in the Americas, a protist parasite that causes human and canine zoonotic visceral leishmaniasis (ZVL)[8]. Domestic dogs are the proven reservoir host[9], though non-reservoir (“dead-end”) hosts, such as chickens and other domestic livestock, are significant blood sources, and assumed to help maintain sand fly populations[10].

The majority of incident human ZVL cases occur in Brazil[8], where the national ZVL control program includes human ZVL case detection and treatment, and reactive IRS of houses and animal sheds within 200m of a newly detected human case[11, 12]. To reduce the canine reservoir population, the program recommends test-and-slaughter or chemotherapeutic treatment of *Leishmania* infected dogs, canine vaccination and/or application of topical insecticides[11]. Despite this extensive arsenal of control tools, there is no apparent decline in human case incidence[13–15]. On the contrary, ZVL has expanded into new geographical regions and into urban settings[15–17]. Thus, sustainable alternative or complimentary methods to combat transmission are needed.

The recent bulk synthesis of the male *Lu*. *longipalpis* sex-aggregation pheromone[18] provides such an opportunity[19]. Male *Lu*. *longipalpis* release the pheromone from abdominal glands, which attracts conspecific males and appetitive females. The resulting leks are formed on or near animal hosts, where the sand flies copulate and the females blood-feed, which results in *L*. *infantum* transmission[20–23]. In field experiments, the synthetic pheromone attracts significantly more *Lu*. *longipalpis* to experimental chicken sheds than to those without the synthetic pheromone[24]. And when co-located with pyrethroid insecticide applied to experimental sheds, it attracts and kills significantly more *Lu*. *longipalpis* compared to untreated control sheds[25]. In a long-lasting controlled release formulation, the pheromone is attractive for up to 3 months[19]. To date, field trials to evaluate the efficacy of this novel lure-and-kill approach to reduce *Leishmania* transmission have not been conducted.

Here we report the results of a stratified cluster randomised trial, conducted in Brazil, to test the efficacy of the synthetic pheromone co-located with a pyrethroid insecticide, to reduce (i) the incidence of *Leishmania* exposure and infection in the canine reservoir; (ii) the abundance of *Lu*. *longipalpis* around households; and (iii) to compare these outcomes in relation to contemporary deployment of deltamethrin-impregnated Scalibor collars fitted to dogs.

## Methods

### Study location

The study was conducted between July 2012 and May 2016 in semi-urban/rural towns and in suburban districts of Araçatuba city (21204011S; 50458883W), located in the administrative region of Araçatuba, N.W. São Paulo state, Brazil. ZVL expanded within this region over the last two decades, where it is now considered endemic[16, 26–29]. The human case incidence was 6.3 per 100,000 with a case fatality rate of 9% recorded in 2011 just prior to the study[27]. This represents the highest human VL incidence within São Paulo state which recorded 2,332 autochthonous cases between 1999 and 2013[28, 30]. Canine seroprevalences in the study region ranged from 12–45% (Superintendência de Controle de Endemias [SUCEN], unpublished data).

### Study design

**Fig 1:**
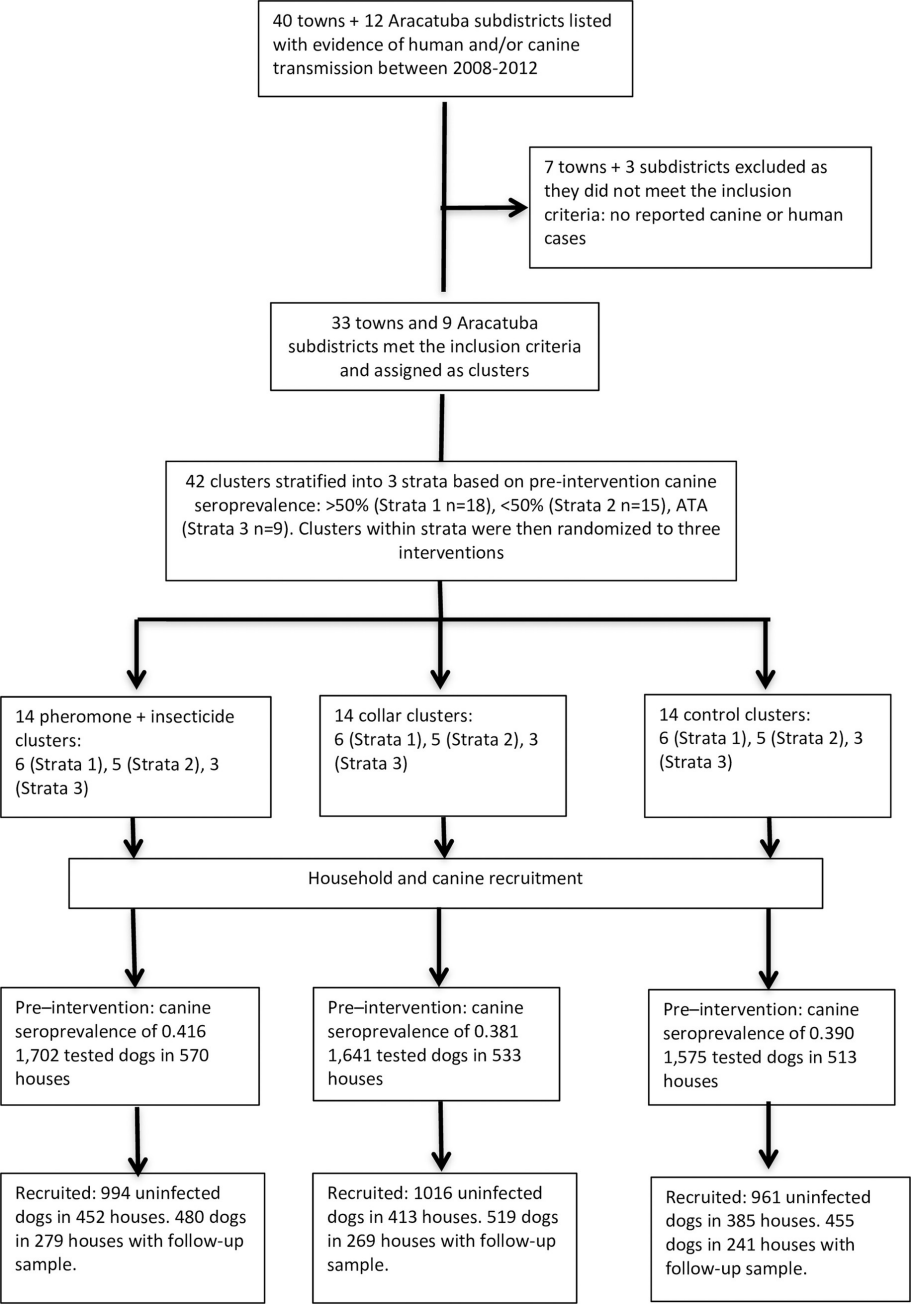
Study design and structure.

The trial was designed as a stratified cluster randomised trial (CRT) where the towns (municipalities), and Araçatuba subdistricts, were designated as independent clusters. Clusters, households and dogs were recruited in a three–step procedure (Fig 1).

### Recruitment

#### Clusters

Forty towns within the Araçatuba administrative region and 12 subdistricts of Araçatuba city, were listed for potential trial inclusion. Cluster inclusion criteria included (i) evidence of recent transmission: at least one confirmed human or/and canine infection within the 4 years prior to the intervention study, by inspection of human case records[30], and canine testing records in 2006–2008 (SUCEN, unpublished data). (ii) That the location was within feasible driving time (1.5 hours) of the trial operations centre in Araçatuba; and (iii) that each cluster was geographically distinct, separated by ≥1 km to minimise any inter-cluster contamination by dispersing *Lu*. *longipalpis* sand fly vectors. Mark-release-recapture studies show ≥97% of *Lu*. *longipalpis* recaptures are within 300m of the release location[21, 31, 32].

Thirty-three municipalities and 9 districts of Araçatuba (42 clusters in total) met these inclusion criteria (Fig 1), being located within an area of approximately 11,250km^2^.

#### Houses and dogs

Local health authorities provided lists of households within clusters for potential recruitment based on criteria that (i) the household maintained at least one chicken (dead-end host) and at least one seronegative dog (defined below) at the time of recruitment; and (ii) the householder(s) and their animals were normally resident. Following consultation with local health authorities, and written permission provided by the municipality health officer, informed written consent was obtained from dog owners to test their dog(s) for anti-*Leishmania* antibodies (described below).

The study experienced substantial loss-to-follow-up (LTF) of dogs and houses primarily due to dogs being lost through mortality or unknown causes and/or households no longer maintaining chickens or eligible dogs. Thus, to fulfil the statistical power requirements, new dogs and houses were recruited between November 2012 and October 2014 (see S1 Table).

### Cluster stratification

Clusters were then stratified, each assigned to one of three strata based on the initial pre-intervention canine seroprevalence within the cluster, of >50% (strata 1: “high” n = 18 clusters), <50% (strata 2: “low” n = 15 clusters), or Araçatuba location (strata 3: “mixed high and low” n = 9 clusters) (Fig 1). Araçatuba clusters were placed in a separate strata as being the regional capital it was considered to have better resources to manage ZVL, knowledge, attitudes and practises (KAP) characteristics, and demographics that could affect transmission dynamics in ways different to in the towns[33].

### Randomisation and treatment allocation

Clusters received one of three treatments, namely (i) synthetic pheromone lure co-located with pyrethroid insecticide; (ii) pyrethroid-impregnated collar fitted to dogs, or (iii) placebo control. These are described below. Within the three defined strata, clusters were ranked in descending order of pre-intervention seroprevalence, and then randomly assigned to one of the three interventions by random number generator in STATA software. All subsequent within-stratum triplet clusters were similarly assigned alternately to intervention groups, resulting in 14 clusters in each treatment arm (Fig 1).

### The interventions

#### Synthetic pheromone lures and insecticide arm

The synthetic pheromone formulation (±-9-methylgermacrene-B [CAS RN: 183158-38-5]) was a copy of the (*S*)-9-methylgermacrene-B pheromone produced by male *Lu*. *longipalpis* from the study region[34]. 10mg of the pheromone was sealed in an 8 cm × 3 cm polythene sachet prototype dispenser designed for slow release (Russell-IPM Ltd. UK), and equivalent to natural pheromone release by 80,000 male *Lu*. *longipalpis* over a 3 month period[19]. Each household received a lure placed within 1m of the main chicken roosting site. Co-located with the pheromone, micro-encapsulated lambda-cyhalothrin (LC-ME) (®Demand 2.5cs, Syngenta, Brazil) was applied at 20mg a.i. m^-2^ to surfaces close to chicken roosting sites using a GUARANY 441–10 compression sprayer (Guarany Industriae Comercio Ltda, Itu, São Paulo, Brazil). Sprayed sites included (i) all available surfaces in and on chicken coops (32.6% of sites), (ii) from ground level up to 3m of the roosting tree, paying special attention to roosting branches (52.5%), or (iii) 3m^2^ (1.5m x 2m) wall surfaces next to ground perches (7.7%), or similar unusual sites (7.2%). Pheromone lures and insecticide were replaced on 9 occasions at an average interval of 91 (S.D. 20.0) days (S2 Table).

#### Insecticide-impregnated collar arm

Scalibor collars (Intervet, MSD Saúde Animal, São Paulo, Brazil) impregnated with 40mg g^-1^ deltamethrin, consisted of a 65cm white polyvinyl chloride strip weighing 25g. Following the manufacturer’s instructions, collars were fitted to dogs ≥3 months old, and expected to reach full activity about 7 days after fitting. All seronegative dogs per house were fitted with a Scalibor collar at baseline. At subsequent recruitment and collaring rounds, all dogs in recruited households were fitted with a collar in order to reduce transmission from positive, potentially infectious, dogs. The veterinary team fitted and replaced any lost collars, promoted their correct use to dog owners, and recorded any adverse reactions and reasons for collar losses. Scalibor collars have an activity period of 5–6 months against sand flies according to the product label, though longer durations of >10 months are experimentally demonstrated[35–37]. Collars were routinely replaced on 5 occasions at an average interval of 182 (S.D. 12.1) days (S2 Table).

#### Control arm

All dogs in control clusters received a placebo collar at recruitment, houses received an identical lure dispenser containing no pheromone, and chicken roosting sites were sprayed with water. For logistic reasons, spraying was limited to two rounds, at baseline and 368 days later (S2 Table). New eligible dogs were fitted with a placebo collar as described; losses of placebo collars or lures were replaced at subsequent dog recruitment rounds (S1 Table).

### Blood sampling and sampling regime

Peripheral blood was collected from dogs by veterinarians by venepuncture onto two replicate Whatman 3MM Chromatography papers (GE Healthcare, 3030–614), which were then dried at ambient temperature, labelled, placed in individual zip plastic bags containing silica gel (Geejay Chemicals), and stored at 4°C until processing. Sera eluted from the filter papers were tested for anti-*Leishmania* antibodies by ELISA. Up to 5mls of blood was also collected into EDTA tubes to harvest leukocytes for molecular detection of *L*. *infantum* parasites by quantitative PCR (qPCR). The laboratory procedures are described in the Supplementary Information file S1 Text.

At baseline, veterinarians scored dogs for eight (non-specific) signs of canine leishmaniasis: alopecia, dermatitis, hyperkeratitis, skin lesions, conjunctivitis, onychogryphosis (excessive nail growth), lunettes, uveitis, and lymphadenopathy (enlarged popliteal lymph nodes). Each sign was scored on a semi-quantitative scale from 0 (absent) to 3 (severe), or 0 to 2 (for onychogryphosis and hyperkeratitis). Scores were then summed to give an overall clinical severity score.

Canine blood samples were collected at baseline to identify potential recruits. Recruited dogs were then sampled and tested again at their final follow-up.

### Trial outcome measures

The primary outcome measure was cluster-level cumulative seroconversion incidence in naïve dogs in each intervention arm compared to in the control arm. The secondary canine outcome measures were *L*. *infantum* parasitological infection incidence, and changes in blood parasite loads (*L*. *infantum* genome equivalents per ml blood buffy coat), confirmed by specific qPCR. The third outcome measure was relative changes in cluster-level household counts of *Lu*. *longipalpis* measured by setting CDC miniature light traps as described below.

Canine clinical condition is not considered a reliable marker of infection incidence following others[38]; signs of canine leishmaniasis are non-specific, thus positive diagnosis requires extensive differential diagnosis, and statistical power was insufficient in this study to rely on changes in advanced canine VL disease.

### Sand fly catches

After the final canine follow-up sample in October 2015, the trial interventions as described above were continued for an additional 7 months until the end of May 2016 to facilitate entomological follow-up. Sand fly sampling was conducted in 42 clusters in 6 approximate quarterly trapping rounds from January 2015 to May 2016 (January/February; April; July/August; October/November in 2015; and January/February; and April/May in 2016). Data from 2 clusters were incomplete and thus excluded from the analyses. The final dataset was generated from 209, 188 and 193 (n = 590) trapping nights in 129, 121 and 113 (n = 363) houses, in 14, 13, and 13 control, pheromone + insecticide and collar intervention clusters, respectively.

Miniature CDC light traps with the light bulb removed were positioned in three locations per household: inside the house preferably in the main bedroom; at the dog sleeping site or kennel entrance; and at the principal chicken roosting site. Each trap was thus associated with the respective host: humans, dogs and chickens. Captured sand flies were sexed and counted under a stereomicroscope. Specific identification was not performed as the vast majority (>98%) of peridomestic sand flies captured in the study region during past and contemporary entomological studies, were confirmed to be *Lu*. *longipalpis* by dissection of spermathecae[39, 40].

### Data collection

Veterinary staff collected details of the dog’s history, and information on any newly acquired dogs, host numbers, and losses of dogs, collars and pheromone lures, by verbal questionnaire to household heads by house-to-house visitation and/or by active telephone contact at least every 3 months.

### Sample size calculations

The sample size was calculated for the primary outcome measure (canine seroconversion incidence) for a two-treatment randomised control trial[41, 42], whereby cluster-level outcomes in collar and in pheromone treated clusters were each compared to the outcomes in the control clusters.

The trial was statistically powered to detect a 50% reduction in *L*. *infantum* seroconversion incidence in naïve dogs after 24 months follow-up with a baseline canine instantaneous annual incidence of 0.6, estimated from canine testing between 2006–2008 in the region (SUCEN, unpublished data) (see Table 1). Calculations were based on a surveyed harmonic mean of 24 dogs per cluster (assuming 1 negative dog recruit per household), with equal numbers of clusters per arm, and coefficient of variation between clusters κ_m_ = 0.40. The latter value was more conservative than κ_m_ = 0.34 estimated from the variation in cluster-level canine seroprevalences (1,701 dogs tested in 42 clusters) at initial trial recruitment. Under these design conditions, 12 clusters per arm were required per intervention arm to achieve a statistical power of 90% with 95% confidence to reject the null hypothesis.

**Table 1:** Serological infection estimates calculated for dogs surveyed prior to trial recruitment, and from canine historical testing records in the same region.

Sample period	Population sample	Force of Infection (FOI) ^1^		Sero-prevalence^2^ (pos/n dogs)
		λ incidence/year (SD), N dogs	ρ recovery/year (SD)	
2012–2014^a^	Control arm	1.14 (0.163), 1,558	1.48 (0.264)	0.610 (961/1575)
	Pheromone arm	1.47 (0.225), 1,693	1.82 (0.335)	0.584 (994/1702)
	Collar arm	1.14 (0.159), 1,631	1.52 (0.265)	0.619 (1016/1641)
	Trial arms combined	1.26 (0.151), 4,882	1.64 (0.235)	0.604 (2,971/4,918)
2006–2008^b^	19 regional towns	0.61 (0.094), 2,304	0.63 (0.121)	0.422 (972/2,304)
2006–2008^c^	20 regional towns			0.451 (4592/10,186)

^1^ FOI estimates were calculated from fitting age-seroprevalence data to an incidence (λ)-recovery (ρ) model.

^2^ Crude seroprevalences estimated including dogs without age records.

^a^ estimates calculated from dogs pre-recruitment resident in the 14 towns (= trial clusters) per intervention arm to which they were subsequently randomly allocated; serological infection detected using the ELISA described in this study.

^b^ FOI estimates calculated from canine samples accompanied by dog age data; serological infection detected using an ELISA kit (EIE-CVL, Bio Manguinhos/Fiocruz-RG, Brazil)

^c^ estimates for the same regional population as in (^b^) but including also test data without dog age records.

To buffer effects of potential loss-to-follow-up (LTF) of clusters and dogs, the number of clusters enrolled was increased to 14 clusters per arm. By the end of the trial no LTF of clusters occurred, though substantial LTF of dogs and houses occurred (see Results). Recalculations at the end of the study, revealed a harmonic mean of 35 dogs followed-up for an average 16.4 months per cluster, with a starting instantaneous incidence of 1.26 year^-1^ measured from age-prevalence data in all recruited dogs prior to being under intervention (Table 1). From these data, the trial design provided 90% statistical power to detect an equivalent 44% reduction in infection incidence between trial arms.

### Statistical analysis

Analysis of the intervention effect on seroconversion and parasite detection incidence were computed using mixed effects binomial complimentary log–log models expressed as incident risk ratios (IRR). Random intercepts for trial clusters were fitted (trial cluster being the higher level of structuring in the data[43]), and log_10_ normalised days under intervention set as the model offset. Similarly, negative binomial mixed effects models were used to test the intervention effects on log_10_ +1 transformed *Leishmania* parasite loads (ml^-1^), and on sand fly numbers.

Complimentary log–log model fits were achieved by Gauss–Hermite numerical adaptive quadrature of the random-effects estimators (quadchk routine in STATA), validated using 16 integration points in model run comparisons to confirm quadrature fitting accuracy; model runs showed ≤0.01% variation in resulting estimates, and were thus considered to be reliable[44].

To measure the effects on canine infection outcomes, models comprised variables describing the trial structure, and variables included *a priori* on the basis that they could affect the trial balance. These were strata (3-levels), baseline canine exposure (baseline anti-*Leishmania* antibody titre), proportion of time (days) under intervention in the bimodally high (December-May) (*cf*. low June -November) sand fly season[45], and household mean numbers of dogs and chickens as surrogates of host odour intensity that may invoke a density-dependent or competing attractant[20, 31, 46].

For analyses of sand fly data, *a priori* covariates included strata, sand fly trapping period (6 levels) reflecting sand fly seasonality, and host abundance at the time of trapping i.e. numbers of people, dogs and chickens associated with each of the 3 trapping locations per household. The model incorporated a cluster term for trial clusters.

The outcomes from these models were considered “unadjusted” effect estimates. Unadjusted estimates were then adjusted on detection of significant model improvement by individual inclusion of additional demographic variables in the model, namely, predominant chicken roosting site category (described above); month and sand fly season (as above) of dog recruitment; dog age at recruitment (median: 24mo.; IQR: 8-48mo.); dog sex; property type (house or small holding) clinical condition score of dog at recruitment (median: 3.1; IQR: 2–4)). Each variable was evaluated for significance by log–likelihood ratio test (LRT) of nested models.

In a secondary analysis, the outcome × strata (3-level) interaction term was tested against the full model to evaluate differential intervention effects between trial strata. In the case that the interaction term was significant with ≥90% probability, individual strata-level effect estimates were further investigated.

The equality of variances in cluster-level dog follow-up time (days) was tested using the Levene's robust test statistic adapted by Brown & Forsythe[47] to provide robust estimators of central tendency (median [W50] and 10% trimmed mean [W10]).

Data were analysed in STATA v.15 (StataCorp LP, College Station, TX).

### Force of infection estimation

The instantaneous incidence (force of infection, FOI) was calculated for dogs serologically tested prior to trial recruitment, by fitting the age-prevalence data to a standard age-incidence-recovery model[48, 49].

To evaluate changes in infection rates from 6–8 years earlier, the FOI was similarly calculated using historical data with age records for 2,304 dogs resident in 19 towns in the same region, sampled in 2006–2008 (SUCEN, unpublished data). Positives were identified on detection of “*L*. *major*-like” promastigote soluble antigens[50] by an ELISA-based kit (EIE-leishmaniose-visceral-canina-Bio-Manguinhos [EIE-LVC], Bio-Manguinhos/Fiocruz- RJ, Brazil). Seroprevalence in the same historical populations was estimated by inclusion of an additional 7,882 dog results that did not have age records (n = 10,186 dogs in total) (see Table 1).

### Data management and masking

Diagnostic results and household questionnaire data were entered into data-checking entry forms designed in ACCESS 2007 relational database by a trained technician, and databases then checked for inconsistencies. Unblinding for final analysis was conducted independently after all dogs had been tested by laboratory staff who were blinded to the cluster treatments and to cluster and household identities through a bar-coding system; all tested sample tubes were bar-coded and results subsequently matched to dog ID bar-codes in the database.

### Ethical considerations

The trial protocols for dogs were approved by the Committee for Ethical Use of Animals (CEUA [FOA-00124-2013]), UNESP, Brazil, and the Animal Welfare and Ethical Approval Body (AWERB, [48723]), University of Warwick, UK. Household questionnaire designs were approval by the Biomedical and Scientific Research Ethics Committee (BSREC, [REGO-2015-1388]), University of Warwick, UK. Informed written consent was obtained from dog owners to sample and fit collars to their dogs, and from the town and district health authorities to conduct the study within their administrative jurisdiction.

## Results

### Pre-enrolment canine infection estimates

**Fig 2:**
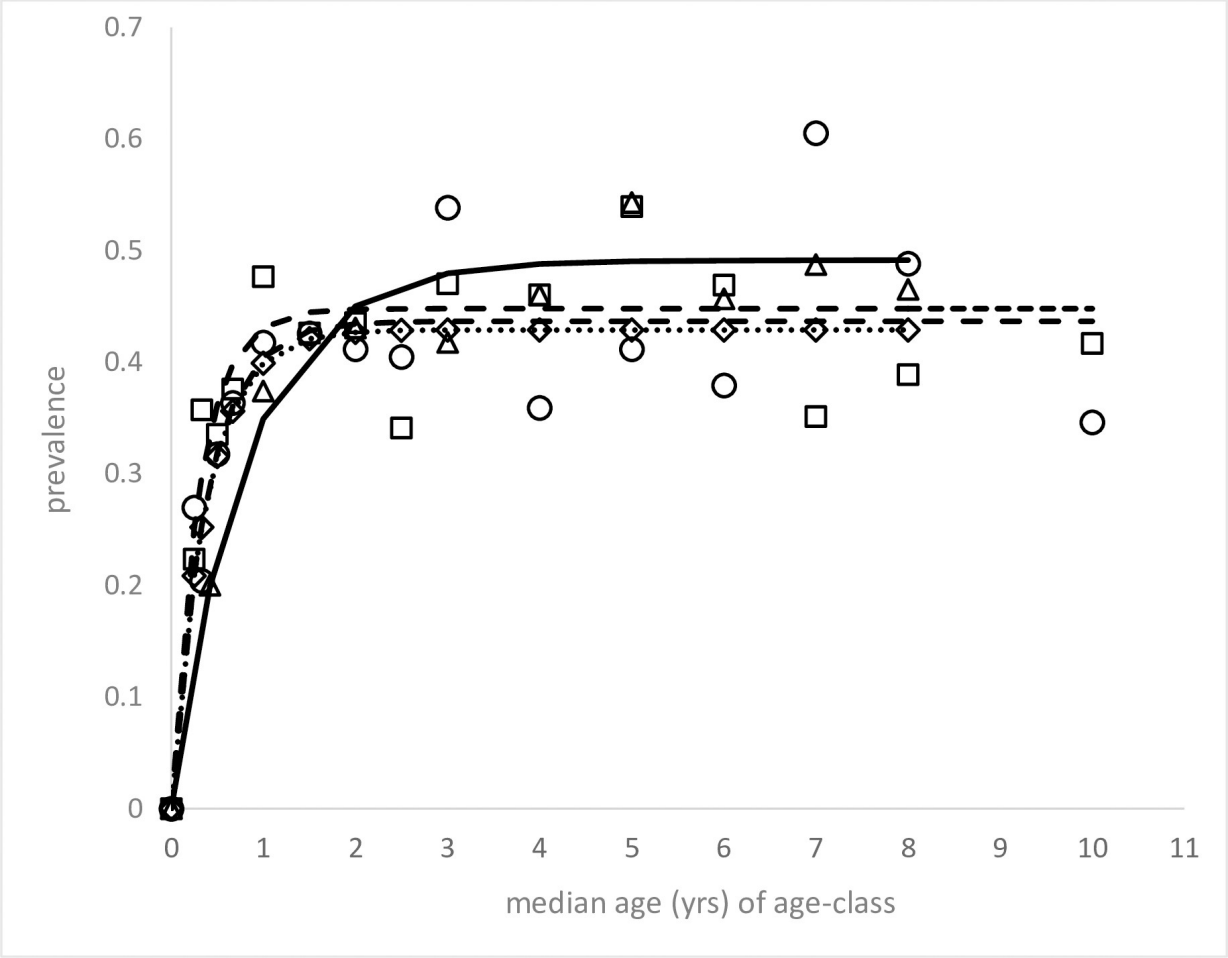
Canine age-seroprevalence data (symbols) fitted to an incidence-recovery model to provide the best fit (lines) from which annual FOI (incidence λ and recovery ρ) were estimated (results shown in Table 1). Data include 4,882 resident dogs in 42 trial clusters sampled prior to recruitment, categorised here according to the intervention arm to which the clusters were subsequently randomised: pheromone (□, ----, n = 1693), collar (◇, ^……^, n = 1631), and control (○,- - - n = 1558) arm. Data also shown for 2,304 dogs resident in 19 towns in the same region sampled in 2006–2008 (△, solid line).

A total 4,918 dogs were serologically tested prior to trial recruitment, of which 2,971 dogs (60%) were seropositive (Table 2). Seroprevalence and FOI estimates were similar between dogs in the three trial arms to which they were subsequently allocated (Table 1; Fig 2). No statistical differences were detected in these infection measures between the trial arms, accounting for the trial structure, date of recruitment, dog age and trial cluster (mecloglog mixed effects model: z<0.89, *P*>0.38). Notably these pre-intervention infection rates were higher than equivalent estimates calculated from canine serosurvey records in the same region conducted a number of years previously (Table 1; Fig 2).

**Table 2:** Summary of the total dogs sampled, recruited and with follow-up sample, according to the trial intervention arm to which the dogs were subsequently allocated.

Intervention arm	Dogs initially sampled for potential recruitment	Seronegative dogs recruited (proportion of sampled)	Negative dogs with follow-up sample (proportion of recruits)
Control	1,575	961 (0.610)	455 (0.473)
Pheromone	1,702	994 (0.584)	480 (0.483)
Collar	1,641	1,016 (0.619)	519 (0.411)
Total	4,918	2,971 (0.604)	1,454 (0.489)

### Dog recruitment and follow-up

The initial enrolment included 630 seronegative dogs under intervention by July-November 2012 (S1 Table). New dogs and houses were recruited between November 2012 and October 2014 (S1 Table). This resulted in a total 2,971 seronegative dogs recruited and placed under the trial interventions, of which, 1,454 (48.9%) dogs, resident in 789 houses across the 42 trial clusters, remained in the study for follow-up testing (Table 2). A median 1 (95% C.I.: 1, 2) seronegative dogs was enrolled per house which did not differ between treatment arms (Poisson: z<0.34, *P*>0.16).

The follow-up cluster population was observed for a *per capita* median 17.1 (95% C.I.s: 15.2, 17.7, n = 455 dogs), 14.7 (95% C.I.s: 14.0, 16.7, n = 480), and 15.2 (95% C.I.s: 13.2, 15.5, n = 519) months under control, pheromone and collar interventions, respectively (S3 Table). Similar fractions of the follow-up times (0.507, 0.454 and 0.416) fell within the seasonally high period of sand fly abundance (December to May) (LRT: χ^2^_(2)_ = 1.08, *P* = 0·58). The variance in cluster-level dog follow-up days were not dissimilar between intervention arms (Levene's W_10_ [df: 2,39] = 1.78, *P* = 0.838; W_50_ [df: 2,39] = 1.60, *P* = 0.853). The epidemiological data for the three intervention arms (Tables 1 and 2; Fig 2), indicated that the randomization process achieved good trial balance.

### Intervention outcomes

#### Seroconversion incidence

**Fig 3:**
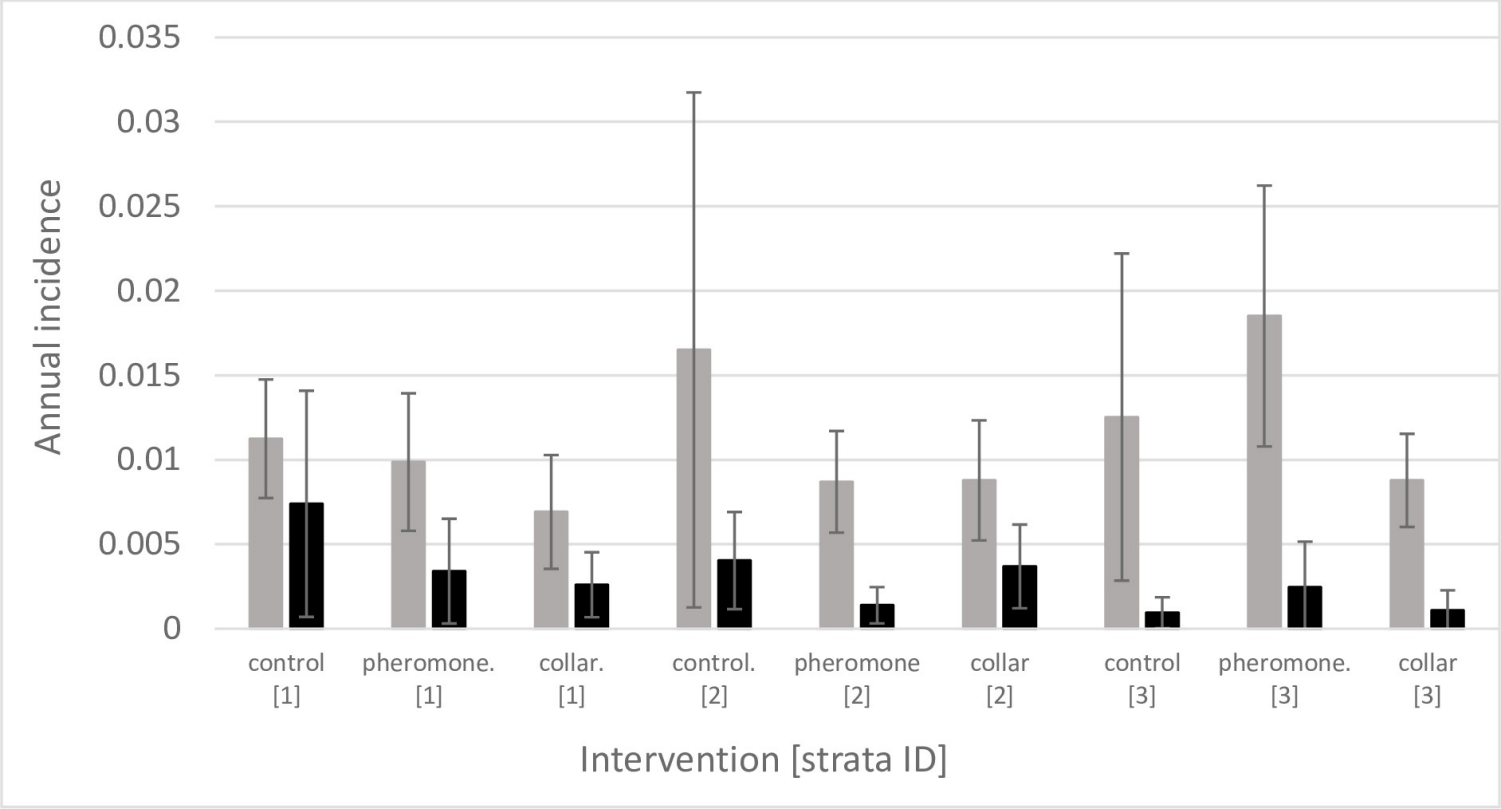
Mean annual incidence (+/- SD) of seroconversion (grey bars) and confirmed parasitological infection (black bars), between trial strata (ID’s 1 to 3) within control, pheromone and collar intervention arms.

Of the seronegative recruits, 225 (49.5%), 217 (45.2%), and 182 (35.1%) in control, pheromone and collar arms respectively, seroconverted by the end of the study. The annual cluster seroconversion incidence varied between the three trial strata within intervention arms (Fig 3; S3 Table).

Accounting for the variables describing the trial structure and follow-up intervals, the unadjusted seroconversion incident risk ratio (IRR) was 0·88 (95% C.I. 0.66, 1.16) in the pheromone arm, and IRR = 0.65 (95% C.I. 0.48, 0.87) in the collar arm, each compared to the control arm (model fit: Wald χ^2^_(8)_ = 31·4, *P*<0.0001) (Table 3).

**Table 3:** Seroconversion incident rate ratio (IRR) amongst dogs under each intervention: unadjusted and adjusted estimates calculated relative to the control arm, by fitting to mixed-effect complimentary log-log models.

Intervention	Unadjusted IRR (95% C.I.s)	N dogs	Adjusted^1^ IRR (95% C.I.s)	N dogs
Pheromone	0.88 (0.662, 1.159), *P* = 0.353	1,443	0.87 (0.660, 1.152) *P* = 0.320	1,322
Collar	0.65 (0.482, 0.868) *P* = 0.004	1,443	0.64 (0.482, 0.856) *P* = 0.003	1,322

^1^ effect modification adjusted by variables significant by LRT: chicken predominant roosting site where pheromone and insecticide were co-located.

Potential adjustment to these estimates was assessed by inclusion of additional demographic variables in the model. Only one significant covariate was identified: the location of the chicken principal roosting site i.e. where the synthetic pheromone + insecticide were co-located (LRT: χ^2^_(3)_ = 9·57, *P* = 0·023). This led to slight modifications of the effect estimates, indicating protection against seroconversion attributed to the pheromone and collar interventions of 13% (95% C.I. 0%, 34.0%), and 36% (95% C.I. 14.4%, 51.8%), respectively (Table 3).

Insecticide treatment of the two most common roosting site categories, low trees (53%), and chicken shelters (33%), were not dissimilar in intervention effect; the significant modification was associated with the third roosting site category, most commonly hollows in the ground, but which represented only 7% of all roost sites. In the latter case insecticide was sprayed on the nearest wall.

Neither intervention resulted in significant changes in the mean log_10_ anti-*Leishmania* antibody units in seroconverted dogs (z<1.45, *P*>0.10).

In a secondary analysis, potential differences in the intervention effects on seroconversion incidence between trial strata were examined. This provided evidence of strata-level variation (strata × treatment interactions, LRT test: χ^2^_(4)_ = 8.34, *P* = 0.078), observed only in the pheromone arm, suggesting a negative impact in Aracatuba city (stratum 3, n = 3 clusters) (IRR = 1.66 [95% C.I.s: 0.971, 2.849], *P* = 0.064) (pheromone arm × stratum 3 interaction: z = 2.36, *P* = 0.018). In contrast, the pheromone effects in strata 1 and 2 (n = 11 town clusters) suggested a protective effect of 27% (95% C.I.s: 0.02%, 46.8%) (IRR = 0.73 [0.532, 0.998], *P* = 0.048).

#### Parasitology

Buffy coat samples from 775 recruited dogs at follow-up were tested for the presence of *Leishmania* kDNA in peripheral blood by qPCR (Table 4). Parasites were detected in 117 (15.1%) of dogs overall; including 20.8% (64/308) of dogs that seroconverted, and 11.4% (53/467) of dogs that did not, by follow-up sample. The latter category of dogs was neither differentially associated with the date or season of recruitment, or their log_10_ follow-up time (fully adjusted model: z>0.049, *P*>0.23), to suggest a predominance of prepatent dogs in the recruited sample.

**Table 4:** Confirmed *Leishmania* infection incidence, tissue parasite loads, and intervention effects in recruited dogs at follow-up.

Intervention arm	qPCR^1^ positive/dogs tested (%)	parasite infection incidence IRR (95% C.I.s)	geometric mean^2^ (95% C.I.) parasite load ml^-1^	parasite load ml^-1^ IRR (95% C.I.s)
control	48/231 (20.8)	referent	18.6 (7.18–47.96)	referent
pheromone	25/246 (10.2)	0·485 (0·251, 0·938) *P* = 0.032	6.48 (0.85–49.35)	0·469 (0·233, 0·946) *P* = 0.034
collars	44/298(14.8)	0·775 (0·425, 1·141) *P* = 0.404	4.82 (1.72–13.54)	0·524 (0·266, 1·03) *P* = 0.062
Total	117/775 (15.1)			

^1^ quantification of *Leishmania* kDNA in canine peripheral blood leukocytes per ml^-1^ by qPCR, standardised to the endogenous control.

^2^ GM calculated in 117 positive dogs with qPCR counts

The percent reduction in the crude number of parasite positive dogs attributed to pheromone and collar interventions at follow-up were 43.3% and 26.1%, respectively (Table 4). Accounting for the trial structure, follow-up periods and covariates in analyses as described above, the levels of protection against confirmed *Leishmania* infection incidence were 51.5% (95% C.I. 6.2%, 74·9%) and 22.5% (95% C.I. 0%, 57·5%), respectively (Table 4). The intervention outcomes did not significantly vary between strata (test of treatment × strata interaction term: LRT: χ^2^_(4)_ = 4.10, *P* = 0·393), nor were effect modifications significant by inclusion of additional demographic variables (LRT: χ^2^_(1–3)_ <2.68, *P*>0·444).

#### *Leishmania* parasite loads

The geometric mean *Leishmania* parasite loads per ml^-1^ in the 117 qPCR positive dogs were highly variable and over-dispersed (Table 4). Relative to control clusters, the pheromone intervention reduced parasite loads by an average 53.1% (95% C.I. 5.4%, 76.7%), and the collar arm by an average 47.6% (95% C.I. 0%, 73.4%) (Table 4). The intervention effects did not significantly vary between strata (test of treatment × strata interaction term: LRT: χ^2^_(4)_ = 0.02, p = 0.905), nor were reductions in parasite loads related to the number of days under intervention (LRT: χ^2^_(4)_ = 0.39, *P* = 0.532), or modified by inclusion of additional demographic variables (LRT: χ^2^_(1–3)_ <0.21, *P*>0·967).

**Fig 4:**
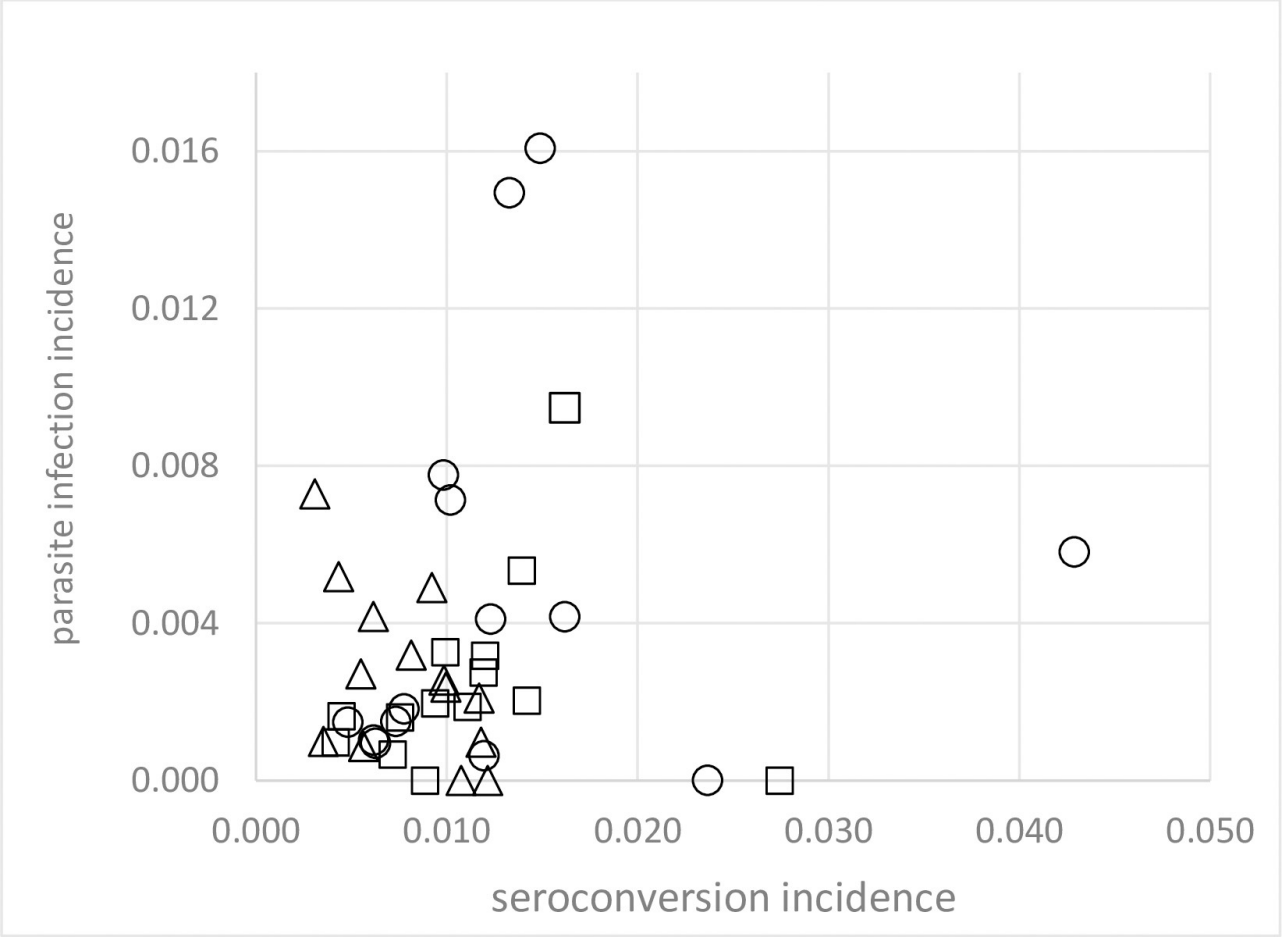
Association between annual incidence estimates of confirmed parasite infection and seroconversion at follow-up in control (○) collar (△) and pheromone (□) intervention clusters.

For the 308 dogs that seroconverted with parasite counts, the log_10_ parasite loads were not correlated with corresponding log_10_ IgG antibody units (Spearman’s r = 0·074, *P* = 0.20); similar non-significant patterns were observed across treatment arms. The annual incidence of confirmed parasitological infection and seroconversion post intervention were also not correlated (Fig 4).

### Sand fly abundance

Complete sand fly trapping records were available for 590 trap nights in 363 houses in 40 trial clusters (S4 Table). The number of trap nights (trapping effort) were similar between intervention arms (t<1.33, *P*>0.19) and between trial strata (t<1.57, *P*>0.124). Relatively few *Lu*. *longipalpis* were captured per house, and only 46% of houses were positive for sand fly capture; which was similar under each intervention (S4 Table).

The pheromone intervention significantly reduced the numbers of female and male sand flies captured at households relative to controls, whereas the collar intervention tended to reduce only the number of females (Table 5). No consistent differences in sand fly captures were observed between the trial strata (test of intervention arm × strata interaction term: z<1.15, *P*>0.248). Inclusion of additional demographic variables did not significantly modify these effect estimates.

**Table 5:** Intervention effects^1^ on household numbers of male and female *Lu*. *longipalpis* sand flies captured across 3 CDC lights traps per house.

Treatment arm	Females IRR (95% C.I.s)	Males IRR (95% C.I.s)
Pheromone	0.51 (0.287, 0.918) *P* = 0.03	0.44 (0.200, 0.974) *P* = 0.04
Collar	0.57 (0.321, 1.007) *P* = 0.05	0.94 (0.460, 1.918) *P* = 0.86

1 estimated from negative binomial mixed effects models including *a priori* predictors.

### Changes in the distribution of vectors at households

Changes in sand fly numbers in CDC traps placed at human, dog and chicken sleeping sites were further examined. In placebo clusters, the majority of *Lu*. *longipalpis* were captured at chicken sleeping sites, with fewer but similar numbers associated with dog sleeping sites, and humans (i.e. inside houses) (Table 6).

**Table 6:** Distribution of household *Lu*. *longipalpis* captured in CDC light traps^1^ located in associated with important blood-source hosts.

Sand flies	Arm	No. on people (proportion of treatment total)	No. on dogs (proportion of treatment total)	No. on chickens (proportion of treatment total)	Total sand flies
Females	Control	34 (0.23)	43 (0.29)	72 (0.48)	149
	Pheromone	20 (0.26)	28 (0.37)	28 (0.37)	76
	Collar	19 (0.22)	17 (0.20)	49 (0.58)	85
Males	Control	45 (0.16)	52 (0.18)	188 (0.66)	285
	Pheromone	31 (0.25)	46 (0.37)	46 (0.37)	123
	Collar	47 (0.15)	37 (0.12)	228 (0.73)	312
All	Control	79 (0.18)	95 (0.22)	260 (0.60)	434
	Pheromone	51 (0.26)	74 (0.37)	74 (0.37)	199
	Collar	66 (0.17)	54 (0.14)	277 (0.70)	397

^1^ In each household, CDC light traps (excluding the bulb) was set inside the house (humans), and outside the house above the sleeping dog, and at the main chicken roosting site.

In the pheromone arm, reductions of 66% (95% C.I. 36%, 81.7%) and 69% (95% C.I. 43.6%, 82.6%) were observed in female and male sand flies captured at the chicken roosting site, being the site of pheromone + insecticide co-location (Table 7). In the collar arm, there was a mean 52% (95% C.I. 0%, 87.9%) reduction in female sand flies at dog trapping sites attributed to collars, although this narrowly failed to reach statistical significance (Table 7). These reductions were not mirrored by significant changes in sand fly numbers at the corresponding alternative trap locations (Table 7).

**Table 7:** Intervention effects on *Lu*. *longipalpis* abundance at host-associated CDC trap sites.

Capture site		Humans IRR (95% C. I.s)	Dogs IRR (95% C. I.s)	Chickens IRR (95% C. I.s)
Female sand flies	Pheromone	0.72 (0.285,1.814) *P* = 0.49	0.71 (0.385, 1.304) *P* = 0.27	0.34 (0.183, 0.640) *P* = 0.001
	Collar	0.55 (0.268, 1.115) *P* = 0.10	0.48 (0.221, 1.052) *P* = 0.07	0.58 (0.296, 1.152) *P* = 0.12
Male sand flies	Pheromone	1.12 (0.451, 2.758) *P* = 0.81	0.80 (0.329, 1.950) *P* = 0.62	0.31 (0.174, 0.564) *P* = 0.001
	Collar	0.93 (0.446, 1.957) *P* = 0.86	0.76 (0.347, 1.641) *P* = 0.48	1.15 (0.564, 2.447) *P* = 0.71

Males made up the majority of captures which was not unexpected (Table 6). The number of female sand flies was positively associated with the number of male flies in the same trap (z = 7.24, *P*<0.0001), but not with the prevailing mean numbers of household chickens or dogs (z<0.49, *P*>0.26). This relationship was not dissimilar across intervention arms (test of intervention arm × male fly number interaction term: z<0.197, *P*>0.53).

### Comparison of the synthetic pheromone versus collar intervention effects

Direct statistical comparisons of the pheromone *versus* collar intervention outcomes (i.e. not compared to the control arm), did not provide evidence of substantial differences between the two interventions. Only in analysis of seroconversion incidence did collars provide an apparent 25.3% (95% C. I.s: 1.2%, 43.4%) additional protection over the pheromone intervention (IRR = 0.747 [95% C.I. 0.566, 0.988], *P* = 0.041). And the pheromone intervention resulted in a 49% (95% C.I. 11%, 66.4%) greater reduction in male *Lu*. *longipalpis* at households compared to in the collar arm (IRR = 0.51 [95% C.I. 0.336, 0.790], *P* = 0.002). Otherwise, no other statistical differences were detected.

## Discussion

The synthetic pheromone intervention reduced the incidence of confirmatory parasitological infection by 52%, and the geometric mean peripheral blood parasite loads by 53%. The same intervention also reduced the household numbers of female *Lu*. *longipalpis* by 49%. These promising outcomes were not mirrored in changes in seroconversion incidence across all trial strata. In the 11 semi-urban town clusters (strata 1 & 2) under this intervention, seroconversion was reduced by an average 27%, whereas on testing the three clusters in Araçatuba city (stratum 3), seroconversion incidence was increased rather than decreased. The latter result on further inspection was specifically attributed to a single Araçatuba treated cluster, in which the annual seroconversion incidence was 0.0274, which was 2.2× the average (0.0126/year) for the three Araçatuba control clusters. The equivalent rates in the other two pheromone-treated clusters (0.0142 and 0.0139/year) were similar to that of controls (S3 Table). Compliance to the synthetic pheromone intervention may have been lower in stratum 3, but we did not detect significant differences (*P*>0.05) in the per lure loss rates between the three strata (range: 0.074–0.107, χ^2^_(2)_ = 5.81), or per collar loss rates (0.156–0.168, χ^2^_(2)_ = 1.16), LTF of recruited dogs (0.527–0.569, χ^2^_(2)_ = 2.9), or the dog recruitment : LTF ratios (0.08–1.28, χ^2^_(2)_ = 3.33). All existing and lost lures were replaced at 3 monthly intervals. There was no further evidence of significant variation in intervention effects between strata in either the pheromone or collar arm. By design, all nine clusters recruited within the regional capital Araçatuba, were assigned to a separate stratum based on the perceived enhanced ZVL control activities and conditions in the city[33].

### Collars

In contrast, the deltamethrin-impregnated collar intervention reduced canine seroconversion incidence by 36% (95% C.I. 14.4%, 51.8%). The attributable reductions in tissue parasite loads of 48% (95% C.I. 0%, 73.4%), and in household female sand flies of 43% (95% C.I. 0%, 67.9%), were indicative, though failed to reach statistical significance (*P*<0.062).

These results were somewhat surprising as the collective studies of Scalibor collars in Brazil[51–55], Europe[56–59], North Africa[60], and central Asia[61] to date, demonstrate their unquestionable impact on *L*. *infantum* transmission, providing a median 56% (IQR: 48.9%- 85.9%; range: 46.9%-100%) protection against canine seroconversion incidence. Moreover, community-wide collar interventions provided 43%-50% protection against human seroconversion and clinical ZVL incidence[61, 62], in addition to reductions in *Lu*. *longipalpis* household abundance[63], and in *Lu*. *longipalpis* infection rates with *L*. *infantum*[51]. Thus, in the current study, the collar intervention arm acted as a positive control for the previously untested synthetic pheromone lure-and-kill method.

The overall protection provided by the collar arm appeared somewhat inferior to that of the pheromone arm in this trial, when each was compared to the control arm. However, a consistent difference in their performance was not detected by direct statistical comparison of the two interventions effect outcome estimates.

### Household sand fly distributions

In control households, the majority of sand flies were captured at chicken roosting sites compared to dog sleeping sites and inside houses. The pheromone intervention diminished female and male sand flies at chicken roosting sites by 49% and 56% respectively. The collar intervention reduced female, but not male, *Lu*. *longipalpis* numbers at dog sleeping locations by 43%. There was no evidence that sand flies were diverted from the treated trap sites to the two alternative untreated host trap sites within households. These results are consistent with the insecticidal effects of the associated interventions, and the success of the purposeful co-location of insecticide at chicken roosting sites. Possible imprecision in the effect estimates arises if the synthetic pheromone recruited additional numbers of sand flies that circumvented traps e.g. through insecticide-induced knockdown or excito-repellency. In this case, it is likely that effect estimates reported here are an underestimate of the true intervention effect. Further experiments are needed to quantify these mechanisms.

### Implications for ZVL control

ZVL control guidelines in Brazil recommend IRS of houses, but also of animal shelters[11], where the majority of *Lu*. *longipalpis* are typically captured[21, 22, 64]. Field studies in north Brazil show sand fly numbers in animals shelters to decrease more or less immediately after insecticide application, but, in parallel, with colonisation of nearby unsprayed sites e.g. household dining huts[65]. Evidenced by additional data[20], the authors of those studies proposed that this shift in vector distribution is a partial consequence of insecticide-induced mortality of male flies causing a decline in pheromone release and recruitment to treated sites. This might increase the relative attractiveness of untreated colonised sites. Based on this rationale therefore, the co-location of synthetic pheromone should maintain sand fly recruitment to insecticide-treated sites. Results supporting this hypothesis demonstrate that the synthetic pheromone can “restore” female and male recruitment to recently sprayed sheds[25]. Indeed, the synthetic pheromone lure tested in this study attracts approximately 24 times more *Lu*. *longipalpis* to chicken sheds compared to sheds without synthetic pheromone[19]. This novel lure-and-kill approach offers a potential improvement to the standard IRS practise against ZVL.

A single 10mg lure synthetic pheromone lure is active for 10–12 weeks[19], and attracts *Lu*. *longipalpis* over distances of 30m in a single night, sufficient to cover the typical urban and more rural household vicinity. The attraction of female *Lu*. *longipalpis* to synthetic pheromone is dose-dependent[23], in line with density-dependent associations between female and male *Lu*. *longipalpis* numbers observed in natural leks / CDC light trap catches (this study; [66, 67]). Chickens are a dead-end (sink) host for *Leishmania*[10], and often the most abundant domestic host in endemic regions[33], hence an obvious location to place the pheromone and insecticide. However, the need for synergistic effects between the synthetic pheromone and host odour to attract *Lu*. *longipalpis*[68] no longer appears critical as confirmed by on-going field studies, thus opening the door for its wider deployment at households without animal hosts. Community-wide experiments are now needed to optimise pheromone doses in different demographic settings. *Lu*. *longipalpis* is a complex of at least four reproductively isolated sibling species, the males of which each produce a different pheromone type[69–71]. The sibling species that produces the (*S*)-9-methylgermacrene-B chemotype is widely distributed in Brazil, and has a geographical range that extends from Argentina to Central America[34, 40, 72–74].

With respect to transmission dynamics, of particular relevance is the observed reductions in tissue *L*. *infantum* parasite loads. This is expected to diminish the canine population’s infectiousness to *Lu*. *longipalpis*. Canine skin, blood, and bone marrow *L*. *infantum* loads correlate with the dogs’ ability to transmit *L*. *infantum* to *Lu*. *longipalpis*[38, 75–78]. Dogs with the highest parasite loads are responsible for the majority of transmission events[38, 76], though not exclusively so[79]. Related to this is the proposal that variation in *Leishmania* metacyclic inoculum from sand fly bites contribute to an individual host’s infection pathology, and subsequent onward transmission potential[80, 81]. Our data indicated that Scalibor collars effectively lowered the over-dispersion in canine population antibody responses to *Lu*. *longipalpis* salivary antigens delivered by sand fly bites, which is indicative of the level of biting exposure. In simple terms, this predicts that a lower fraction of dogs would receive high density *L*. *infantum* challenge under the trialled intervention.

The FOI estimates suggest an increase in canine transmission from 2006–2008 to the trial period (Table 1), which is supported by independent reports of the rise and spread of canine infection, and *Lu*. *longipalpis* abundance, across São Paulo state[13, 16]. Together with current upward trends in human ZVL burdens in Brazil[13–15], the need for sustainable vector control is clear. The MoH policy of culling seropositive dogs continues to be unpopular amongst dog owners[82], and in the Brazilian setting, dog collars, along with the registered canine vaccine Leish-Tec, and anti-*Leishmania* chemotherapy treatment options, may be too costly and/or perceived insufficiently effective, to achieve community-level compliance[83, 84]. Scalibor collar labels indicate 5–6 months effective duration, though collar losses from dogs are variably high (range: 0.6–8.2% per month)[52, 54–57, 59, 61, 62], necessitating replacement, particularly in regions of year-round transmission. In this trial, collar losses were 7.5% (95% C.I.: 6.5, 8.6) per month, compared to pheromone lure loss of 2.7% (95% C.I.: 0.26, 5.2) per month. On bulk synthesis, the potential unit cost of a pheromone lure is likely to be substantially lower than the cost of collars, vaccines or canine chemotherapeutic treatment.

## Conclusions

Manipulation of vector behaviour is an often overlooked but important component of effective vector control. The collective results of this study indicate a potential role of the lure-and-kill approach to combat *L*. *infantum* transmission in Brazil. The protective effects were not dissimilar to those of the insecticide-impregnated collars, although the confidence intervals around all effect estimates were broad. Notwithstanding, it is reasonable to consider that robustly designed deployment of the lure-and-kill strategy could result in public and veterinary health benefits similar to those globally reported for the Scalibor collars. In order to maximise the synthetic pheromone efficacy, complimentary studies are underway to inform best practice for community-level deployment.

## Supporting information

S1 Text Laboratory methods. (DOCX)

S1 Table Numbers of dogs recruited to each intervention arm per period of the study. (DOCX)

S2 Table Intervention dates and intervals for the three intervention arms. (DOCX)

S3 Table Number of conversions to seropositive and parasite positive per cluster across strata and intervention arms in recruited dogs. Crude annual incidence shown for both measures.
(DOCX)

S4 Table Summary of sand fly trapping effort and capture success in the intervention arms. (DOCX)
