# Supplementary material for: Sand fly synthetic sex-aggregation pheromone co-located with insecticide reduces the incidence of infection in the canine reservoir of visceral leishmaniasis: A stratified cluster randomised trial
Source: PLoS Negl Trop Dis. 2019 Oct 25;13(10):e0007767. doi: 10.1371/journal.pntd.0007767 (PMC6834291; doi:10.1371/journal.pntd.0007767)
Supplement: S1 Text — (DOCX) [file pntd.0007767.s001.docx]

S1. Laboratory methods

Detection of anti-*Leishmania* antibody by ELISA

*Leishmania* IgG antibodies were eluted from 6 x 3mm punched blood spots from the Whatman 3MM filter paper as suggested by[1]. Eluted antibodies were transferred to 2D barcoded protein Cryogenic vial matrix plate (Thermo Scientific Nunc, CRB-374-014F) for fridge storage at +4^o^C for the immediate use or freezer storage -20 ^o^C for longer term storage.

For the antigen preparation, crude *Leishmania* antigen (CLA) was produced from promastigotes cultures of *Leishmania infantum* (ITMAP263/MON-1) as described[2]. The CLA protein concentration was measured using BCA Protein Assay Kit (Thermo scientific, UK) by following the manufacturer’s recommendations, and diluted to reach a concentration of 1μg/100μl per well for coating ELISA plates. Linbro flat bottom microplates (MP Bio Cat No 097633105) coated with CLA were re-suspended in a carbonate coating buffer (pH 9.6) at the final concentration of 1µg per well and incubated overnight at 4 ^o^C. ELISA assays were performed according following[3].

For standardization, positive endemic canine control sera (dogs D17 and C15 from northern Brazil) were titrated 2-fold (15 positive standards) on every plate from 1/200 to 1/3,276,800. Negative endemic control sera from Brazilian dogs (C14 and A84) were also titrated 2-fold on every plate from 1/200 to 1/16,000. Following exploratory analysis, it was decided to titrate test sera at 1/200. Negative control serum from non-endemic UK and endemic Sao Paulo city, Brazil, canine populations were also included on every plate in addition to two blank cards in 2 wells, and 2 wells containing no sample. In order to measure background signals.

The absorbance of the plates was read at 405nm using a Wallac Victor2 1420 Multilabel Counter (PerkinElmer, USA). The optical density (O.D) of the standards and test samples was converted to arbitrary units/ml following others (e.g. [3] where the standard curve was linearised by plotting the optical density (O.D) of the standards minus the O.D of the blank, against the log_10_ unit values of the standards. The standardised unit values of the test samples were calculated based on the linear curve produced by 1:10 dilution of highest positive control sample.

ELISA cut-off values

Antibody log_10_ unit values >2.861 were considered positive for *Leishmania* infection representing the mid-point between the lower 99% confidence limit value (CL) of the median value for 188 endemic dogs with confirmed parasite infection (DNA detection in blood leukocytes by qPCR), and the upper 99% CL of 1,512 endemic parasite negative dogs that showed no clinical signs VL, where the CLs were calculated using a distribution-free binomial approximation method[4]. Control dog samples were collected from the endemic Aracatuba canine population. Comparable threshold values were indicated from data of 48 endemic negative dogs from Crete, Greece, that showed no clinical signs of infection or immunological or molecular evidence of infection from testing six clinical samples by ELISA and qPCR (L. Calvo-Bado et al. unpublished data).

Extraction of DNA from buffy coat

EDTA vacutainer tubes were centrifuged at 3000rpm for 10 minutes at 4°C, and the buffy coat carefully removed. DNA was extracted from buffy coat using Chemagic Blood Spot Kit LH (Chemagic, PerkinElmer, USA) by following the manufacturer’s recommendations with some modifications. The DNA extraction was carried out using semi-automated liquid handling system JANUS (PerkinElmer, USA). The eluted DNA (approximately 195 µl) was transferred into a 2D barcoded Martix plate (Fisher scientific, UK) for short term storage at +4^o^C and longer term storage at -20^o^C. The matrix plate was scanned using VisionMate SR Avision AVA6 Plus (Thermo Scientific, UK) and matched to dog ID template for record keeping and data analysis.

Cultivation of *Leishmania infantum* and preparation of qPCR standards

*Leishmania infantum* promastigotes (ITAMP-263DD8) were cultivated in M199 medium for 3-5 days as described[2]. The number of *L. infantum* cells per ml were enumerated by hemocytometer. DNA was extracted from *L. infantum* cells using NucleoSpin Tissue Kit (Macherey-Nagel, GmbH and Co, Duren, Germany) by following the manufacturer’s recommendations with some modifications. Serial dilution of the neat DNA starting from 10^8^ cells^-ml^ to 10^-3^ cells^-ml^ was performed to produce 8 standards for qPCR.

Quantitative PCR

*L. infantum* infection confirmation and parasite load per gram of ear tissue were determined by quantitative PCR using the Applied Biosystems 7500 Fast real-time detection system (Applied Biosystems, Warrington, UK). All qPCR reactions were performed in duplicate using the Automated Liquid Handling System (JANUS, PerkinElmer, USA). Each qPCR reaction contained 6.5 µl 2X ABI Master Mix (Applied Biosystems, Warrington, UK), 1.17 µl each of *Leishmania* forward primer (5’ – AACTTTTCTGGTCCTCCGGGTAG – 3’) and *Leishmania* reverse primer (5’ – ACCCCCAGTTTTCCGCC – 3’) (10 pmol ml-1), 0.325 µl *Leishmania* probe (5’ – 6FAM – CCAGGCTCGAAGTTGTTGCTGCCC – BBQ) (10 pmol ml-1), 2.835 µl nuclease free water and 2 µl of template DNA. DNA was tested at 1:10 dilution; samples that yielded negative amplification were further diluted to 1:50 and retested to reduce the likelihood of inhibition. A non-template control (nuclease free water) was included in triplicate in all PCR reactions. The reaction was carried out under the following conditions: one cycle at 50 ^o^C for 2 min, one cycle at 95 ^o^C for 10 min, followed by 40 cycles at 95 ^o^C for 15s and 55 ^o^C for 1 min. Absolute quantification of *L. infantum* was estimated based on comparison of the C_t_ values to the standard curve constructed from 10-fold serial dilutions of DNA extracted from *L.* *infantum* (strain ITAMP-263DD8) promastigotes and ranged between 10^5^ to 0.01 parasite equivalent/mg which were run in triplicate in each qPCR plate.

Endogenous control

A Taqman assay was designed to amplify a 187-bp product from the canine housekeeping β-actin gene to control for DNA extraction efficiency. β-actin was quantified in a separate qPCR reaction to the TaqMan *Leishmania* assay, in the same manner using 10 pmol ml-1 each forward primer ACTB F 5’-CCTgCggCATCCATgAAA-3’, reverse primer ACTB R 5’ ggggTgCgATgATCTTgATCTT-3’, and β-actin probe ACTB TM 5’-AggACCTCTATgCCAACACAgTgCTgT-3’ (FAM-labelled TaqMan, TIB MOLBIOL, Germany). Standard curves were prepared using known copy numbers of the β-actin gene (10^5^ to 10^1^ per reaction) from a plasmid containing the 187-bp β-actin fragment. This amplicon was cloned into TOPO PCR 2.1 (Invitrogen) cloning vector, *E. coli* transformed and plasmid purified. Parasites contain two copies of the β-actin gene. From these data, *Leishmania* parasite loads per ml of buffy coat were standardised by dividing the crude number of *Leishmania* parasites detected by qPCR by the number of β-actin genes detected per sample.

1. Corran PH, Cook J, Lynch C, Leendertse H, Manjurano A, Griffin J, et al. Dried blood spots as a source of anti-malarial antibodies for epidemiological studies. Malaria Journal. 2008;7. doi: 10.1186/1475-2875-7-195. PubMed PMID: WOS:000260391800001.

2. Coderre JA, Beverley SM, Schimke RT, Santi DV. Overproduction of a bifunctional thymidylate synthetase-dihydrofolate reductase and DNA amplificaton in methotrexate-resistant Leishmania tropica. Proceedings of the National Academy of Sciences of the United States of America-Biological Sciences. 1983;80(8):2132-6. doi: 10.1073/pnas.80.8.2132. PubMed PMID: WOS:A1983QM10300008.

3. Quinnell RJ, Courtenay O, Garcez L, Dye C. The epidemiology of canine leishmaniasis: Transmission rates estimated from a cohort study in Amazonian Brazil. Parasitology. 1997;115(2):143-56. PubMed PMID: BIOSIS:PREV199799711066.

4. Mood AM, Graybill, F.A. Introduction to the Theory of Statistics. New York: McGraw-Hill Book Company; 1963.
