# Supplementary material for: Sand fly synthetic sex-aggregation pheromone co-located with insecticide reduces the incidence of infection in the canine reservoir of visceral leishmaniasis: A stratified cluster randomised trial
Source: PLoS Negl Trop Dis. 2019 Oct 25;13(10):e0007767. doi: 10.1371/journal.pntd.0007767 (PMC6834291; doi:10.1371/journal.pntd.0007767)
Supplement: S1 Table — (DOCX) [file pntd.0007767.s002.docx]

S1. Numbers of dogs recruited with follow-up sample per intervention arm

| recruitment round | date range | Intervention arm | | | total |
| --- | --- | --- | --- | --- | --- |
|  |  | control | pheromone | collar |  |
| 1 | 7/7/12 - 5/11/12 | 203 | 210 | 217 | 630 |
| 2 | 8/11/12 - 30/1/13 | 29 | 25 | 13 | 67 |
| 3 | 14/2/13 - 3/5/13 | 20 | 57 | 43 | 120 |
| 4 | 17/5/13 - 8/8/13 | 38 | 34 | 86 | 158 |
| 5 | 12/8/13 - 10/10/13 | 98 | 79 | 59 | 236 |
| 6 | 20/10/13 - 30/1/14 | 17 | 12 | 30 | 59 |
| 7 | 6/2/14 - 16/4/14 | 6 | 10 | 20 | 36 |
| 8 | 7/5/14 - 7/8/14 | 8 | 25 | 16 | 49 |
| 9 | 11/8/14 - 31/10/14 | 36 | 28 | 35 | 99 |
|  |  |  |  |  |  |
|  | Totals | 455 | 480 | 519 | 1454 |
