# Supplementary material for: Sand fly synthetic sex-aggregation pheromone co-located with insecticide reduces the incidence of infection in the canine reservoir of visceral leishmaniasis: A stratified cluster randomised trial
Source: PLoS Negl Trop Dis. 2019 Oct 25;13(10):e0007767. doi: 10.1371/journal.pntd.0007767 (PMC6834291; doi:10.1371/journal.pntd.0007767)
Supplement: S2 Table — (DOCX) [file pntd.0007767.s003.docx]

S2. Intervention dates and intervals for the three trial arms.

| intervention round | pheromone + insecticide | collars | placebo control |
| --- | --- | --- | --- |
| 1 | 6/11/12 - 7/12/12 | 8/11/12 - 14/12/12 | 14/1/13 - 7/2/13 |
| 2 | 14/2/13 - 4/4/13 |  |  |
| 3 | 6/5/13 - 11/6/13 | 2/5/13 - 13/6/13 |  |
| 4 | 5/8/13 - 10/9/13 |  |  |
| 5 | 5/10/13 - 29/11/13 | 22/10/13 - 29/11/13 | 13/1/14 - 14/2/14 |
| 6 | 1/2/14 - 17/4/14 |  |  |
| 7 | 7/5/14 - 6/6/14 | 20/5/14 - 5/6/14 |  |
| 8 | 4/8/14 - 12/9/14 |  |  |
| 9 | 27/10/14 - 17/12/14 | 3/11/14 - 12/12/14 |  |
|  |  |  |  |
| average (SD) interval (days) from round mid-point date | 91 (20.0) | 182 (12.1) | 368 |
