# Supplementary material for: Sand fly synthetic sex-aggregation pheromone co-located with insecticide reduces the incidence of infection in the canine reservoir of visceral leishmaniasis: A stratified cluster randomised trial
Source: PLoS Negl Trop Dis. 2019 Oct 25;13(10):e0007767. doi: 10.1371/journal.pntd.0007767 (PMC6834291; doi:10.1371/journal.pntd.0007767)
Supplement: S3 Table — Crude annual incidence shown for both measures. (DOCX) [file pntd.0007767.s004.docx]

S3. Number of negative recruited dogs that converted to seropositive and parasite positive by follow-up per cluster across strata and intervention arms. Crude annual incidence shown for both measures.

| Strata / intervention arm | cluster ID | seropositive /n | parasite positive /n | total dog follow-up years | seroconversion incidence /year | parasite infection incidence /year |
| --- | --- | --- | --- | --- | --- | --- |
| **strata 1** |  |  |  |  |  |  |
| Control | 18 | 11/44 | 2/25 | 51.8 | 0.0048 | 0.0015 |
| Control | 21 | 8/18 | 3/9 | 33.5 | 0.0133 | 0.0149 |
| Control | 34 | 23/35 | 1/25 | 55.0 | 0.0119 | 0.0006 |
| Control | 37 | 18/30 | 10/14 | 40.2 | 0.0149 | 0.0161 |
| Control | 40 | 24/41 | 8/20 | 57.4 | 0.0102 | 0.0071 |
| Control | 42 | 15/30 | 4/17 | 40.6 | 0.0123 | 0.0041 |
|  |  |  |  |  |  |  |
| Pheromone | 19 | 31/67 | 4/32 | 111.3 | 0.0042 | 0.0010 |
| Pheromone | 20 | 6/24 | 1/15 | 26.7 | 0.0094 | 0.0020 |
| Pheromone | 26 | 9/25 | 4/12 | 22.2 | 0.0162 | 0.0095 |
| Pheromone | 30 | 10/31 | 2/22 | 42.7 | 0.0075 | 0.0016 |
| Pheromone | 31 | 21/32 | 4/19 | 54.7 | 0.0120 | 0.0032 |
| Pheromone | 32 | 10/28 | 2/13 | 36.0 | 0.0099 | 0.0033 |
|  |  |  |  |  |  |  |
| Collar | 13 | 8/27 | 2/20 | 30.1 | 0.0098 | 0.0026 |
| Collar | 14 | 6/36 | 1/11 | 38.6 | 0.0043 | 0.0052 |
| Collar | 15 | 3/14 | 0/9 | 17.6 | 0.0121 | 0.0000 |
| Collar | 23 | 22/62 | 3/26 | 100.3 | 0.0035 | 0.0010 |
| Collar | 25 | 14/42 | 6/28 | 54.2 | 0.0062 | 0.0042 |
| Collar | 39 | 8/33 | 3/18 | 44.2 | 0.0055 | 0.0027 |
| **strata 2** |  |  |  |  |  |  |
| Control | 11 | 28/42 | 9/17 | 67.8 | 0.0098 | 0.0078 |
| Control | 29 | 19/43 | 2/22 | 70.6 | 0.0063 | 0.0009 |
| Control | 35 | 8/13 | 1/7 | 14.4 | 0.0429 | 0.0058 |
| Control | 41 | 15/27 | 3/16 | 34.3 | 0.0162 | 0.0042 |
| Control | 43 | 16/36 | 2/17 | 60.5 | 0.0073 | 0.0015 |
|  |  |  |  |  |  |  |
| Pheromone | 10 | 20/39 | 1/18 | 46.3 | 0.0111 | 0.0019 |
| Pheromone | 12 | 16/47 | 4/34 | 76.0 | 0.0045 | 0.0016 |
| Pheromone | 16 | 18/44 | 1/21 | 57.2 | 0.0071 | 0.0007 |
| Pheromone | 22 | 11/28 | 1/9 | 33.0 | 0.0119 | 0.0028 |
| Pheromone | 36 | 8/24 | 0/13 | 37.6 | 0.0089 | 0.0000 |
|  |  |  |  |  |  |  |
| Collar | 17 | 17/31 | 1/19 | 46.5 | 0.0118 | 0.0010 |
| Collar | 24 | 18/37 | 6/23 | 52.8 | 0.0092 | 0.0049 |
| Collar | 27 | 10/26 | 2/19 | 32.9 | 0.0117 | 0.0021 |
| Collar | 28 | 22/46 | 6/28 | 58.9 | 0.0081 | 0.0032 |
| Collar | 38 | 7/44 | 10/30 | 51.5 | 0.0031 | 0.0073 |
|  |  |  |  |  |  |  |
| **strata 3** |  |  |  |  |  |  |
| Control | 44 | 10/35 | 1/19 | 46.3 | 0.0062 | 0.0010 |
| Control | 45 | 13/23 | 0/9 | 23.9 | 0.0237 | 0.0000 |
| Control | 46 | 17/38 | 2/15 | 57.7 | 0.0078 | 0.0018 |
|  |  |  |  |  |  |  |
| Pheromone | 49 | 18/31 | 2/15 | 40.9 | 0.0142 | 0.0020 |
| Pheromone | 50 | 27/40 | 2/12 | 48.5 | 0.0139 | 0.0053 |
| Pheromone | 52 | 12/20 | 0/11 | 21.9 | 0.0274 | 0.0000 |
|  |  |  |  |  |  |  |
| Collar | 47 | 11/27 | 0/19 | 37.9 | 0.0108 | 0.0000 |
| Collar | 51 | 16/40 | 2/17 | 40.2 | 0.0100 | 0.0024 |
| Collar | 53 | 20/54 | 2/31 | 65.8 | 0.0056 | 0.0009 |
