# Supplementary material for: Sand fly synthetic sex-aggregation pheromone co-located with insecticide reduces the incidence of infection in the canine reservoir of visceral leishmaniasis: A stratified cluster randomised trial
Source: PLoS Negl Trop Dis. 2019 Oct 25;13(10):e0007767. doi: 10.1371/journal.pntd.0007767 (PMC6834291; doi:10.1371/journal.pntd.0007767)
Supplement: S4 Table — (DOCX) [file pntd.0007767.s005.docx]

S4. Summary of sand fly trapping effort and capture success in the trial arms.

| Intervention arm (number clusters sampled) | Prop. of trap nights sand flies captured (N) | Prop. of houses where sand flies were captured (N) | Total  female sand flies | Total male sand flies | Geometric mean^1^ (95% C. I.) per cluster |
| --- | --- | --- | --- | --- | --- |
| Control  (14) | 0.45 (209) | 0.47 (129) | 149 | 285 | 3.64 (0.56, 12.80) |
| Pheromone (13) | 0.36 (188) | 0.45 (121) | 76 | 123 | 2.47 (0.33, 8.04) |
| Collar  (13) | 0.41 (193) | 0.47 (113) | 85 | 312 | 3.18 (0.48, 10.87) |
| Totals  (40) | 0.40 (590) | 0.46 (363) | 310 | 720 |  |

^1^ Williams mean includes zero counts transformed by adding 1 to all counts, taking the geometric mean, then subtracting 1 from the values.
